# High‐Rate Alkaline Water Electrolysis at Industrially Relevant Conditions Enabled by Superaerophobic Electrode Assembly

**DOI:** 10.1002/advs.202206180

**Published:** 2022-12-11

**Authors:** Lingjiao Li, Petrus C. M. Laan, Xiaoyu Yan, Xiaojuan Cao, Martijn J. Mekkering, Kai Zhao, Le Ke, Xiaoyi Jiang, Xiaoyu Wu, Lijun Li, Longjian Xue, Zhiping Wang, Gadi Rothenberg, Ning Yan

**Affiliations:** ^1^ School of Physics and Technology Wuhan University Wuhan 430072 P. R. China; ^2^ Van't Hoff Institute for Molecular Sciences (HIMS) University of Amsterdam Amsterdam 1098XH The Netherlands; ^3^ School of Power and Mechanical Engineering Wuhan University Wuhan 430072 P. R. China

**Keywords:** alkaline water electrolysis, industrial conditions, hydrophilicity, bifunctional catalysis, bubbles

## Abstract

Alkaline water electrolysis (AWE) is among the most developed technologies for green hydrogen generation. Despite the tremendous achievements in boosting the catalytic activity of the electrode, the operating current density of modern water electrolyzers is yet much lower than the emerging approaches such as the proton‐exchange membrane water electrolysis (PEMWE). One of the dominant hindering factors is the high overpotentials induced by the gas bubbles. Herein, the bubble dynamics via creating the superaerophobic electrode assembly is optimized. The patterned Co‐Ni phosphide/spinel oxide heterostructure shows complete wetting of water droplet with fast spreading time (≈300 ms) whereas complete underwater bubble repelling with 180° contact angle is achieved. Besides, the current collector/electrode interface is also modified by coating with aerophobic hydroxide on Ti current collector. Thus, in the zero‐gap water electrolyzer test, a current density of 3.5 A cm^−2^ is obtained at 2.25 V and 85 °C in 6 m KOH, which is comparable with the state‐of‐the‐art PEMWE using Pt‐group metal catalyst. No major performance degradation or materials deterioration is observed after 330 h test. This approach reveals the importance of bubble management in modern AWE, offering a promising solution toward high‐rate water electrolysis.

## Graphical abstract

The superaerophobic electrode assembly enables the optimization of bubble dynamics in the alkaline electrolyzer, significantly decreasing the ohmic loss during water splitting at high current densities. The resulting zero‐gap water electrolyzer delivers a current density of 3.5 A cm^−2^ at 2.25 V and 85 °C in 6 m KOH.

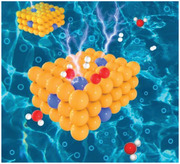

## 1 Introduction

Producing green hydrogen using water electrolysis and renewable energy is essential for tomorrow's decarbonized economy.^[^ ^1^, ^2^, ^3^, ^4^ ^]^ Compared with the proton‐exchange membrane water electrolyzer (PEMWE), alkaline water electrolyzer (AWE) is particularly advantageous as it enables the use of materials comprising affordable earth‐abundant elements.^[^ ^5^, ^6^, ^7^, ^8^ ^]^ This technology has a long history of industrial deployment, hence enjoying relatively low operating expense (OPEX).^[^ ^9^, ^10^ ^]^ Nevertheless, conventional AWE delivers much lower operating current densities (<0.5 A cm^−2^) in comparison with that of PEMWE (>2 A cm^−2^).^[^ ^11^, ^12^ ^]^ Therefore, developing new materials and methods to boost the hydrogen production rate in AWE becomes a central focus in both academic and industrial research.

One of the key hindering factors is the huge polarization loss associated with the bubbles adhering to the electrode.^[^ ^13^, ^14^ ^]^ At high current densities, it is estimated that such bubble‐induced overpotential is comparable to or even greater than the activation overpotential of the state‐of‐the‐art catalysts. The increase of the ohmic loss is the most dominant effect.^[^ ^15^ ^]^ Theoretically, the ohmic area‐specific resistance (ASR) of a classic zero‐gap AWE in 30 wt% KOH electrolyte at 80 °C is ≈0.13 Ω cm^2^ using a 0.5 mm‐thick Zirfon separator, which is comparable to that of the Nafion‐based PEMWE (≈0.16 Ω cm^2^).^[^ ^16^, ^17^ ^]^ In practice, however, this value often surpasses 0.3 Ω cm^2^ due to the “gas blanket” on the electrode and separator, impairing the transport of ions.^[^ ^18^ ^]^ Besides, additional overpotential loss can originate from the bubble “formation‐departure” cycle which brings complicated local convection of the electrolyte. The blocked active sites by the bubble also causes extra energy loss at high current density operation.^[^ ^19^, ^20^, ^21^ ^]^

There are indeed various engineering strategies of optimizing the bubble dynamics such as the “flow‐through‐the‐electrode” configuration,^[^ ^22^ ^]^ bubble‐free operation using capillary diffusion,^[^ ^23^ ^]^ and facilitated mass transport by ultrasound^[^ ^24^ ^]^ or magnetic field.^[^ ^25^ ^]^ From the perspective of materials science, finding superaerophobic electrode surface seems to be a more facile approach as it does not involve the architecture change of the conventional AWE.^[^ ^26^, ^27^, ^28^ ^]^ Recently, tremendous progress has been made on developing active and superaerophobic catalysts. Yet the underwater bubble contact angles of them are often in the range of 150–170°, it remains challenging to further enhance the aerophobicity to minimize the overpotential loss.^[^ ^29^, ^30^, ^31^, ^32^ ^]^ Furthermore, despite their superior performance at low reaction rates (tens to hundreds of mA cm^–2^) and in half‐cell reactions, such catalysts have been rarely tested in zero‐gap AWE or at industrially relevant conditions. It is yet unclear whether the optimized bubble dynamics can indeed dramatically decrease the overpotential at high reaction rate (>2 A cm^−2^).

In this work, we created the superaerophobic electrode assembly by simultaneously optimizing the electrode and the electrode/current collector interface. The developed cobalt‐nickel phosphide/oxide heterostructure was a superaerophobic‐superhydrophilic electrode that was bifunctionally active for both hydrogen evolution (HER) and oxygen evolution reactions (OER). The complete wetting of water droplet with fast spreading time was achieved whereas complete underwater bubble repelling led to 180° contact angle of air bubble. The zero‐gap AWE integrated with this catalyst and a superaerophobic current collector delivered a current density of 3.5 A cm^−2^ with 2.25 V bias at 85 °C. This approach reveals the significance of bubble management over the performance of AWE, offering a promising solution toward high‐rate water electrolysis.

## 2 Superaerophobic‐Superhydrophilic Heterostructure

**Scheme 1:**
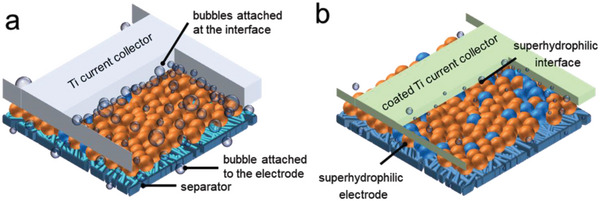
The schematic comparison of bubble evolution in conventional and aerophobic electrode assembly.

**Scheme** **1**a depicts the bubble evolution on the electrode of a conventional alkaline water electrolyzer. It is well documented that, in addition to the normal electrode surface, a substantial portion of bubbles are also generated at the interface of the current collector (bipolar plate in a stack) and electrode. Therefore, in this work, we simultaneously manipulated the aerophobicity of both the electrode and the interface as shown in Scheme 1b.

**Figure 1:**
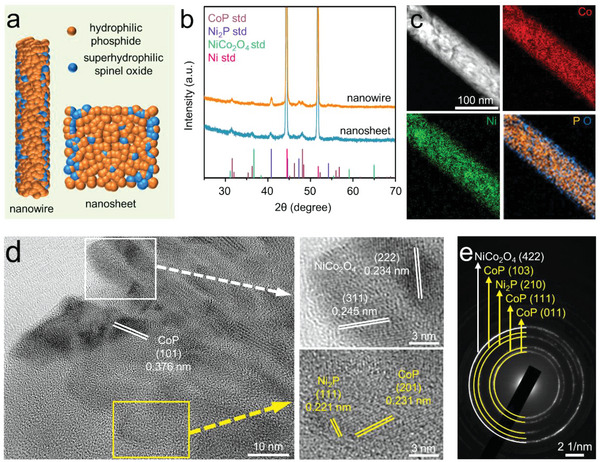
a) The schematics of hybrid catalyst with phosphide and spinel oxide domains; b) XRD patterns of nanostructured hybrid catalyst; c) dark‐field TEM micrograph and the corresponding EDX elemental mappings of Ni, Co and P/O; d) HRTEM micrograph and e) selected area electron diffraction (SAED) graph of hybrid catalyst.

Generally, both the aerophobicity and hydrophilicity of materials are governed by the topography and chemical composition of the surface. Thus, we first designed a new electrode material comprising Ni_2_P‐CoP phosphide domains and NiCo_2_O_4_ spinel oxide domains (see **Figure** **1**a). The phosphide is hydrophilic with proven activities in both OER and HER^[^ ^33^, ^34^ ^]^; the spinel oxide is an excellent OER catalyst with superhydrophilicity in nature after surface oxidation. Importantly, the heterostructure promise to have synergistic effects toward higher electro‐activity.^[^ ^35^ ^]^ Both nanowire and nanosheet structures were prepared on a Ni sheet by the phosphidation of Ni‐Co hydroxide in controlled atmosphere (see the Supporting Information for details). Ni_2_P, CoP, and Ni‐Co spinel oxides were all identified in the X‐ray diffraction (XRD) patterns (Figure 1b). From the transmission electron microscope (TEM) analysis, we noticed the homogenous distribution of both Co and Ni over the entire nanostructure; yet elemental mappings from the energy‐dispersive X‐ray spectroscopy (EDX) suggested the local segregation of phosphorus and oxygen in the hybrid structure (see Figure 1c). In the high‐resolution TEM micrograph in Figure 1d, we observed two types of domains with distinct d‐spacings. The P‐rich domains indeed contained Ni_2_P and CoP whereas the O‐rich domains were consisted of NiCo_2_O_4_. This conclusion was in accordance with the selected area electron diffraction (SAED) pattern in Figure 1e and was aligned with the surface analysis results using X‐ray photoelectron spectroscopy (XPS) in Figure S6 (Supporting Information).

**Figure 2:**
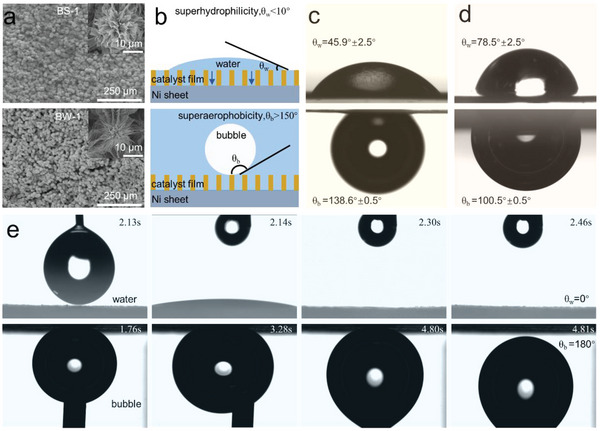
a) SEM images of BS‐1 and BW‐1, additional microstructural images of other analogues were shown in the supporting information; b) the schematics of superhydrophilic and superaerophobic surface; optical images of water and air bubble on the surface of c) R‐P; d) NS; and e) BS‐1.

We then optimized the surface morphologies of the hybrid catalyst via tuning the synthetic conditions (see the Supporting Information). Seven different patterns comprising nanowires (NW), “nanowire bundles” (denoted as BW‐1, BW‐2, and BW‐3), and “nanosheet bundles” (denoted as BS‐1, BS‐2, and BS‐3). All bundles were ≈20 µm in diameter, yet the wire/sheet densities varied in each category (low to high density from 1 to 3). Their scanning electron microscope (SEM) images were shown in **Figure** **2**a and Figure S8 (Supporting Information). The prepared patterns showed different degrees of wettability. A superhydrophilic surface shows a low water droplet contact angle (<10°) while a superaerophobic surface is featured by as a high bubble contact angle (>150°) underwater (see the schematic in Figure 2b). The regular phosphide surface (R‐P) was indeed hydrophilic with a water contact angle (*θ*_w_) of 45.9 ± 2.5°, significantly lower than the industrial catalyst of pristine Ni surface (*θ*_w_ = 78.5 ± 2.5°). The incorporation of spinel oxide indeed boosted the hydrophilicity as all hybrid surfaces were superhydrophilic, showing the complete wetting with *θ*_w_ = 0°. This means the gas‐solid interfacial tension (*γ*_gs_) is larger than the sum of liquid–solid (*γ*_ls_) and gas–liquid (*γ*_gl_) interfacial tensions.

To quantitatively compare the wetting dynamics of the superhydrophilic surfaces with 0° contact angle, we used the spread time (Δ*t*) as the descriptor which was defined by the time interval between the droplet contact timepoint (*t*_ws_) and the finish timepoint of the complete wetting (*t*_wf_) as shown by the schematic in Figure S10 (Supporting Information).^[^ ^36^ ^]^

(1) $$\Delta t=t_{\mathrm{wf}}-t_{\mathrm{ws}}$$

The spreading was driven by the interfacial tensions parallel to the solid surface at the line of contact, the net force per unit length of the triple interface along the solid surface (*F*_h_) can be defined by:

(2) $$F_{h}=\gamma_{\mathrm{gs}}\;-\gamma_{\mathrm{ls}\;}-\;\gamma_{\mathrm{gl}\;}\cos\theta^{\prime }$$

where the angle *θ*′ is the instantaneous contact angle that changes as the triple interface advances toward complete wetting (*θ*′ = 0°). A smaller Δ*t* reflects the larger average acceleration of the spreading and consequently the larger average *F*_h_ according to Newton's laws of motion. The more rapid spreading of water (hence more rapid recession of the gas phase) is beneficial for the removal of the readily formed bubble on the electrode. Figure S11 and Videos [Link], [Link], [Link], [Link], [Link], [Link], [Link], [Link], [Link] (Supporting Information) compared the Δ*t* of all the patterned surfaces. It appeared that the density of nanostructure on the surface had a positive correlation with Δ*t*: Both BW‐3 (0.97 s) and BS‐3 (1.96 s) showed the largest Δ*t* while BW‐1 (0.80 s) and BS‐1 (0.33 s) showed the minimum in each category.

Similarly, the underwater bubble contact angle (*θ*_b_) of pristine Ni was 100.5 ± 0.5°, smaller than that of smooth phosphide surface (*θ*_b_ = 138.6 ± 0.5°). The patterned surfaces were much more aerophobic: the bubble cannot even adhere to the surface and moved away with the needle tip after touching the surface. This scenario remained when increasing the contact area by pushing the bubble against the surface to introduce the deformation (see Figure 2e). Thus *θ*_b_ = 180° was achieved for all the hybrid surfaces. These results from such captive bubble measurement well matched the abovementioned results using sessile drop method as *θ*_w_ = 180°−*θ*_b_. From these wettability results, we deduced that the integration of spinel oxide enabled superhydrophilicity whereas surface topology optimization affected “spreading dynamics” and could contribute to faster removal of gas bubbles.

## 3 Bifunctional Activities of Patterned Heterostructure

**Figure 3:**
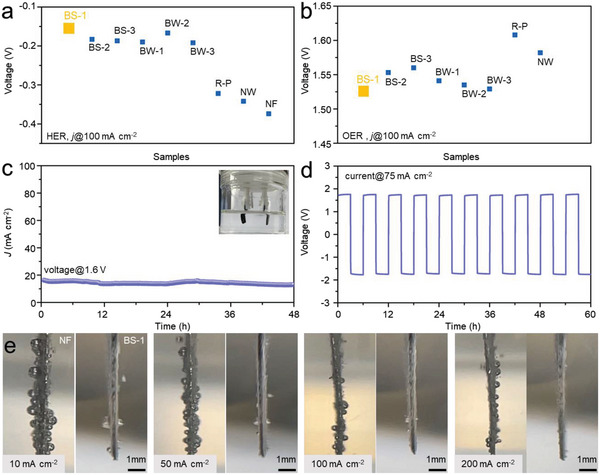
a) HER and b) OER performances of all catalysts at 100 mA cm^−2^ in 1 m KOH; overall water splitting was performed using bifunctional catalyst BS‐1 at c) 1.6 V constant bias and d) ± 75 mA cm^−2^ current density; e) surface bubbles comparison of NF and BS‐1 electrode at different HER current densities.

We then constructed the same patterns on Ni foam and evaluated their HER and OER activities using the standard 3‐electrode setup. The catalyst mass loadings for all samples were controlled in the range of 3.5 ± 1.5 mg cm^−2^ (see Figure S13, Supporting Information). **Figure** **3**a,b compares the overpotentials of various catalysts at 100 mA cm^−2^ for HER and OER, respectively. BS‐1 performed the best for both reactions, showing 158 mV (HER) and 293 mV (OER) overpotentials. In particular, the HER activity of the hybrid catalyst was not undermined by introducing the spinel oxide into the surface, which is comparable with pure phosphide reported in the literature.^[^ ^37^, ^38^ ^]^ The detailed polarization curves, Tafel slopes and electrochemical active surface area (ECSA) of different catalysts are summarized in Table S1 and Figures S14 and S15 (Supporting Information). We can deduce that the excellent activity of BS‐1 is not determined by the mass loading of active species, yet is closely related to the high ECSA of the catalyst.

Based on the overall performance on the surface wettability and catalytic activities in HER and OER, we selected BS‐1 as the candidate electrode material for overall water splitting test. We initially examined the HER and OER stabilities of BS‐1 electrode using cyclic voltammetry (CV). After 8000 redox cycles, no apparent activity degradation can be observed from the voltammograms (see Figure S17, Supporting Information). The performance increase in HER might be ascribed to the reduction of oxidized Ni/Co species. The consequently formed metallic phase are also active for water reduction.^[^ ^39^ ^]^ In the 48 h water electrolysis with both electrodes employed by BS‐1 catalysts, the voltage stabilized at ≈1.6 V at a constant current density of 15 mA cm^−2^ (Figure 3c), which was among the best comparing with the state‐of‐the‐art bifunctional catalysts (see in Table S2, Supporting Information). No potential degradation was observed during the cyclic current polarization change (±75 mA cm^−2^) as shown in Figure 3d. These results confirmed that the BS‐1 pattern was bifunctionally active and redox stable, further proving the excellent OER/HER activity of phosphide‐spinel oxide hybrid which was comparable with or better than the well‐documented phosphide catalysts.

The hydrodynamic behavior of H_2_ and O_2_ bubbles on the superaerophobic electrode was also monitored from 10 to 200 mA cm^−2^. On commercial Ni HER catalyst, small bubbles easily aggregated to larger ones. This was because the adhesive force of bubble on the electrode was strong, bubble departure would occur until the buoyancy force was larger than this force. Thus, large bubbles with size > 0.1 mm were observed on Ni electrode at all the studied current densities. The high bubble coverage on electrode might undermine the electrolyte transfer and increase the polarization loss (vide infra).^[^ ^21^, ^40^ ^]^ Conversely, the superaerophobic nature of BS‐1 substantially minimized the adhesive force of bubbles on the surface. Small bubbles left the surface “prematurely” as the buoyancy force was already larger than the adhesive force, creating a “white bubble cloud” around the electrode even when the current density was low (also see Video S10, Supporting Information). This implied that the residence time of such bubbles on the electrode is significantly shorter than those with larger bubbles. The faster formation‐departure kinetics implied more intense micro‐convection of electrolyte and the longer period of operation time of electrode at “high‐electrolyte‐coverage” status (see the schematic illustration in Figure S20, Supporting Information), both of which will decrease the electrode overpotential.^[^ ^21^, ^41^ ^]^

## 4 Water Splitting at Industrially Relevant Conditions

**Figure 4:**
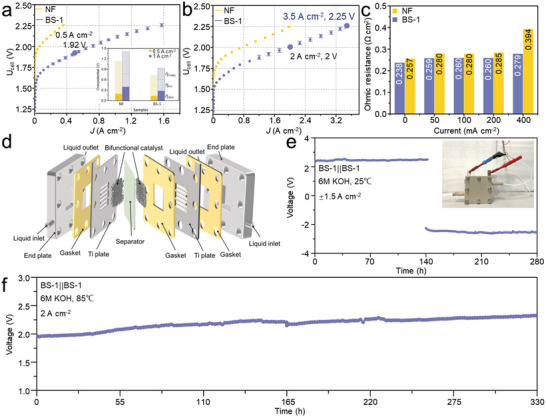
Overall water splitting using a zero‐gap AWE at a) 25 °C and b) 85 °C; c) ohmic ASR variation as a function of current densities; d) the schematic of the modified zero‐gap electrolyzer; stability tests at e) 25 °C and f) 85 °C.

Using BS‐1 as both electrodes, we finally assembled a zero‐gap AWE, simulating the industrial practice. Both anolyte and catholyte (6 m KOH solution) were circulated to ensure stable OH^−^ concentrations in anode/cathode compartments and to prevent the accumulation of bubbles inside. To create the superaerophobic interface between electrode and current collector, the Ti current collector was coated by cobalt hydroxide via electrochemical deposition which was detailed in the Supporting Information. To avoid the overheating of the electrolyzer, the maximum applied voltage was limited at ≈2.25 V. At 25 °C, a current density of 0.5 A cm^−2^ was achieved at the voltage of 1.92 V in BS‐1 AWE (**Figure** **4**a). This performance is comparable with typical zero‐gap AWEs in the literature that worked at room temperature.^[^ ^42^, ^43^ ^]^ We have also separated the overpotentials contributed by electrode kinetics, ohmic resistance and mass transfer at the current densities of 0.5 and 1 A cm^−2^ (see the inset in Figure 4a). The concentration polarization increased when the current density rose to 1 A cm^−2^ in the cell with NF electrode, accounting for nearly half of the total polarization loss. However, in addition to the decreased concentration overpotential in BS‐1 cell relative to that of the NF cell, there is no significant concentration overpotential increase when the current density was increased from 0.5 to 1 A cm^−2^. Both observations confirmed that the superaerophobic nature of the BS‐1 electrode has substantially facilitated the mass transport and minimized the associated overpotential loss.

At 85 °C, our cell demonstrated much better performance: 2.0 A cm^−2^ was obtained at 2 V and the current density increased to 3.5 A cm^−2^ at 2.25 V. This high current density is substantially higher than those reported in the previous work and is even comparable with PEMWE cell employed with noble catalysts. A detailed comparison can be found in Table S3 (Supporting Information). We have also determined the Faradaic efficiency (FE) of the cell under the current density of 1 A cm^−2^. The drainage method was used to collect the generated H_2_ and O_2_ during the electrolysis. The volumes were compared with the theoretical values and shown in Figure S23 (Supporting Information). The obtained Faradaic efficiencies for the generation H_2_ and O_2_ were 99.6% and 98.9%, respectively. High‐purity gas (>98.2%) was obtained in both anodic and cathodic chambers at different current densities (see Figure S24, Supporting Information).

In the electrochemical impedance spectroscopy (EIS) with the cell biased at 0, 50, 100, 200, and 400 mA cm^−2^, we noticed that the area specific resistance (ASR) of the cell decreased progressively. However, the ohmic portion of Ni cell increased by ≈53% (0.137 Ω cm^2^) compared with that obtained at the open‐circuit voltage (OCV) as shown in Figure 4c. Yet, BS‐1 cell only showed an increase of ≈17% (0.041 Ω cm^2^). We attributed this ohmic resistance increase mainly to the bubbles adhering to the electrodes and the neighboring diaphragm surface. Thus, ohmic overpotential loss at high current densities became less significant on superhydrophilic BS‐1 electrode. Nevertheless, it should be noted that the ohmic ASR at operation (0.279 Ω cm^2^) is yet significantly higher than the theoretical value (≈0.1 Ω cm^2^) calculated based on the conductivity of the diaphragm. Further investigation of the bubble dynamics might be helpful in minimizing the ohmic loss. We have also fitted the Nyquist plots and shown the equivalent circuits in Figure S25 (Supporting Information). The charge transfer resistance associated with the catalytic activity for BS‐1 was apparently lower than that of NF, which was mainly due to the higher activity of the hybrid catalyst. The Warburg element, which is linked to the diffusion process, was unfortunately not prominent. This is due to the perturbation induced by the bubbles evolution which made the accurate collection of data points at low frequency difficult.

To demonstrate the advantage of the superaerophobic current collector/electrode interface, we designed a control experiment using a high‐speed camera to observe the interface between BS‐1 electrode and a current collector (see the schematic in Figure S26, Supporting Information). When the current collector is coated by polytetrafluoroethylene (PTFE), although the generated small bubbles left BS‐1 surface rapidly despite of the current density value; the free bubbles in the electrolyte could attach to the hydrophobic PTFE surface. Theoretically, attached bubbles would continuously collide and merge with the incoming small bubbles, grow bigger and leave the surface. Yet, we noticed that this bubble growth is rather slow: the collision of free bubbles with the stationary bubble is elastic in many cases that does not lead to the coalescence of two bubbles (see Figure S27 and Video S11, Supporting Information). Therefore, the bubble residence time on non‐electrode hydrophobic surface is rather long, which might be detrimental to the performance of AWE. To the contrary, the superaerophobic interface between BS‐1 and cobalt hydroxide coated current did not show any accommodation of bubbles (Figure S27, Supporting Information).

In the longevity test, we first investigated the stability of BS‐1 cell at 25 °C. A constant current density of 1.5 A cm^−2^ was applied for 280 h, the recorded voltage was stabilized at 2.26 V with negligible degradation. Importantly, when the current polarity was swapped at 140 h, the cell worked equally well thereafter, suggesting the excellent redox stability of the bifunctional hybrid electrode. This feature might be industrially important as most commercial AWE are vulnerable at shut‐down/stand‐by conditions when destructive phase transition and dissolution of the electrode might occur.^[^ ^44^ ^]^ At 85 °C, we biased the BS‐1 cell at 2 A cm^−2^. The initial voltage was 1.98 V, close to that shown in the *I*–*V* curve, and finally stabilized at ≈2.2 V. No major voltage degradation was recorded after 330 h. We also characterized both electrodes after the stability test. Albeit that electrode corrosion indeed occurred, most of the surface remained intact and the bundled sheet pattern of the electrode was maintained for both HER and OER electrodes. In the XPS analysis, the nickel and cobalt phosphide phases remained in the spent catalyst from the cathode, which were believed as the active phase for the HER (see Figure S31, Supporting Information). For the anode catalyst, large amount of oxides and oxyhydroxides were observed for both Ni and Co, which were widely documented as the active species for the OER.

## 5 Conclusion

In conclusion, the patterned Co‐Ni phosphide/spinel oxide hybrid showed superaerophobicity with complete wetting of water droplet and complete underwater bubble repelling with 180° contact angle. The AWE with both current collector and electrode coated by this material demonstrated excellent performance in terms of high current density of 3.5 A cm^−2^ at 85 °C and 2.25 V in 6 m KOH electrolyte. No major performance degradation or materials deterioration was observed after 330 h test. This approach reveals the importance of bubble management in modern AWE, offering a promising solution toward high‐rate water electrolysis.

## Conflict of Interest

The authors declare no conflict of interest.

## Supporting information

Supporting Information

Supplemental Video 1

Supplemental Video 2

Supplemental Video 3

Supplemental Video 4

Supplemental Video 5

Supplemental Video 6

Supplemental Video 7

Supplemental Video 8

Supplemental Video 9

Supplemental Video 10

Supplemental Video 11

## Data Availability

The data that support the findings of this study are available from the corresponding author upon reasonable request.
