# Supplementary material for: High‐Rate Alkaline Water Electrolysis at Industrially Relevant Conditions Enabled by Superaerophobic Electrode Assembly
Source: Adv Sci (Weinh). 2022 Dec 11;10(4):2206180. doi: 10.1002/advs.202206180 (PMC9896032; doi:10.1002/advs.202206180)
Supplement: Supplementary file 1 — Supporting Information [file ADVS-10-2206180-s010.pdf]

ADVANCED SCIENCE
Open Access

Supporting Information

for *Adv. Sci.*, DOI 10.1002/advs.202206180

High-Rate Alkaline Water Electrolysis at Industrially Relevant Conditions Enabled by Superaerophobic Electrode Assembly

*Lingjiao Li, Petrus C. M. Laan, Xiaoyu Yan, Xiaojuan Cao, Martijn J. Mekkering, Kai Zhao, Le Ke, Xiaoyi Jiang, Xiaoyu Wu, Lijun Li, Longjian Xue, Zhiping Wang, Gadi Rothenberg and Ning Yan**

Supplementary Information

High-rate alkaline water electrolysis at industrially-relevant conditions enabled by superaerophobic electrode assembly

Lingjiao Li[1], Petrus C. M. Laan[2], Xiaoyu Yan[1], Xiaojuan Cao[1], Martijn Mekkering[2], Kai Zhao[1], Le Ke[1], Xiaoyi Jiang[1], Xiaoyu Wu[1], Lijun Li[3], Longjian Xue[3], Zhiping Wang[1], Gadi Rothenberg[2], Ning Yan[1,2]

1 School of Physics and Technology, Wuhan University, Wuhan, China.

2 Van't Hoff Institute for Molecular Sciences (HIMS), University of Amsterdam,

3 School of Power and Machinery, Wuhan University, Wuhan, China.

E-mail: ning.yan@whu.edu.cn

Methods

Materials.

Ammonium fluoride (NH_4F, 96.0%), urea ($CO(NH_2)_2$, 99.0%), sodium hypophosphite monohydrate ($NaH_2PO_2 \cdot H_2O$, 99.0%), ethanol (C_2H_5OH, 99.7%), acetone (C_3H_6O, 99.5%), hydrochloric acid (HCl), and potassium hydroxide (KOH, .385.0%), were purchased from Sinopharm Group, China. Cobalt nitrate hexahydrate ($Co(NO_3)_2 \cdot 6H_2O$, 99%) was purchased from Aladdin. All chemicals were used without further purification. Nickel sheet (NS, thickness: 0.1 mm) and nickel foam (NF, thickness: 1 mm, porosity: ~95%) were offered by Suzhou Sinero Technology Co., Ltd. Polyethersulfone (PES) membrane was obtained from Hangzhou Cobetter Filter Equipment Co., Ltd. Deionized water was used to prepare all solutions and electrolyte.

Preparation of cobalt-nickel phosphide/spinel oxide hybrid.

Preparation of Co, Ni hydroxides. A piece of NS (1×2 cm^2) or NF (1×2 cm^2) was sequentially cleaned by ultrasonication with hydrochloric acid, acetone, ethanol and deionized water. The cleaned substrate was then put into an autoclave with a polytetrafluoroethylene (PTFE) lining (25 ml). 15 ml aqueous solution containing $Co(NO_3)_2 \cdot 6H_2O$ (0.375 mmol), NH_4F (1.5 mmol) and $CO(NH_2)_2$ (1.875 mmol) was then added. The hydrothermal synthesis of hydroxide was carried out at 120 °C for 12 h. Cobalt hydroxide was coated on Ti current collector by electrodeposition which was detailed in our previous work.

Preparation of Co-Ni phosphide/spinel oxide hybrid. The prepared oxide patterns on NS or NF (1 piece) were phosphidated in the atmosphere of PH_3 containing Ar (flow rate: 40 ml/min) at 300 ^oC using our previous approach described elsewhere[1]. The degree of phosphidation was controlled by varying the mass of $NaH_2PO_2 \cdot H_2O$, which was the P source, in the tube. 0.2 g of $NaH_2PO_2 \cdot H_2O$ was identified as the optimal loading to partially phosphidate hydroxide into phosphide while the residual hydroxide thermally converted into spinel oxide, the chemical equation as follows:

$$4Co(OH)_2 + 2Ni(OH)_2 + O_2 \rightarrow 2NiCo_2O_4 + 6H_2O$$

Structural Characterization.

X-ray diffraction (XRD) was carried out using the diffractometer by Rigaku (Cu Kα radiation).

Scanning electron microscopy (SEM, HITACHI S-4800) and transmission electron microscopy (TEM, JEM-F200) with energy dispersive X-ray spectroscopy (EDX) were used to characterize the morphology and the local chemical composition. X-ray photoelectron spectroscopy (XPS) was performed on an X-ray photoelectron spectrometer (Thermo Scientific, USA) with monochromatic Al Kα X-ray source (hv=1486.6 eV). An OCA25 contact angle meter (Dataphysics, Germany) was used to measure the water droplet contact angle and the underwater bubble contact angle. The volumes of both water droplet and air bubble were 3 μl. The dynamic behavior of bubbles at the hydrophilic-hydrophobic interface was characterized using a high-speed camera (Fuhuang Junda, 5KF10-M, 3600 fps).

Electrochemical test.

The catalytic activity of each electrode samples was determined using a three-electrode configuration connected to a potentiostat (Autolab pgstat M204) at room temperature in 1M KOH (pH~14) solution. A graphite rod was used as the counter electrode for hydrogen evolution reaction (HER) and oxygen evolution reaction (OER) testing; a standard Hg/HgO electrode was used as the reference electrode. All potentials applied were converted to the reversible hydrogen electrode (RHE) using the equation: $E_{RHE} = E_{Hg/HgO} + 0.098 + 0.0591 \times pH$. Linear sweep voltammetry (LSV) was tested at 1 mV/s between -0.875 V and 0.125 V (vs. RHE), and between 0.925 V and 1.925 V (vs. RHE) for HER and OER, respectively. Prior to each measurement, 50 times of CV cycles was applied. The resistance (R) of the system was then determined using electrochemical impedance spectroscopy (EIS). Post-iR compensation was applied using 90% of the determined R.

The overpotential (η) for HER was calculated as η (V) = 0 – E_{RHE} and that for OER was calculated as η (V) = E_{RHE} – 1.23 V. The Tafel formula (η = a + b log (j), η is the overpotential, b is the Tafel slope, j is the current density) was used to calculate the Tafel slope. The electrochemically active surface area (ECSA) of the prepared catalyst was determined by measuring the double-layer capacitance (C_{dl}) of the non-Faraday region. Generally, cyclic voltammetry scans were performed at scan rates from 15 to 35 mV s^{-1} in the potential range of 0.025 to 0.075 V versus RHE. Other than specified, all the polarization resistance was determined at the overpotential of 75 mV using EIS in the frequency range from 10^{-2} to 10^{5} Hz. In the two-electrode setup for overall water electrolysis, the as-prepared bifunctional catalysts were used as both the anode and cathode.

The zero-gap electrolyzer comprises two PEEK endplates, gaskets and two titanium plates with flow channels as the current collector. Porous polyethersulfone (PES) with a thickness of 0.12 mm and the pore diameter was 0.22 μm, which was used as the separator. 6 M KOH solution was used as both the anolyte and catholyte. The circulation of the electrolyte was enabled by the peristaltic pump at 40 rpm (flow rate: 10 ml/min) and the inner diameter of the hose was 3 mm. The electrolyte containers and the electrolyzer was submerged in a closed flowing water bath at 85 °C to avoid overheating of the electrolyzer during operation. No iR compensation was applied. The cell potential E_{cell} consists of the following parts: $E_{cell} = E_{rev} + \eta_{ohm} + \eta_{kin} + \eta_{mass}$. The standard reversible potential E_{rev} is 1.23 V at normal temperature and pressure. The ohmic overpotential η_{ohm} is obtained from the EIS spectra. The kinetic overpotential η_{kin} is extracted from the Tafel model. We assumed that the OER controlled the kinetic overpotential of the whole cell following the method reported in the literature. The exchange current density j_0 is calculated by the extrapolation of Tafel curve, $\eta_{kin} = b \times \log (j/j_0)$. Mass transfer overpotential η_{mass} is obtained by subtracting the reversible potential, ohmic overpotential and kinetic overpotential from the cell potential. The purity of hydrogen and oxygen generated was analyzed by gas chromatography (GC9790 plus, Fuli, China).

Faradaic efficiency measurement.

Faradaic efficiency (FE) was measured in a zero-gap electrolyzer using 6 M KOH as electrolyte. The bifunctional catalyst BS-1 was used as cathode and anode. Chronopotential test was performed at a current density of 1 A cm^{-2} to produce hydrogen and oxygen continuously. The generated H_2 and O_2 were collected by drainage method and recorded every 5 minutes. Faradaic efficiency (FE) was calculated according to the following formula:

$$FE = \frac{n(gas)_{measured}}{n(gas)_{theoretical}} = \frac{PV(gas)_{measured}/RT}{Q/zF}$$

Where P is the pressure, V is the volume of gas generated, R is the gas constant, T is the temperature, Q = It is the charge, z is the number of stoichiometric charges (2 electrons per H_2 molecule, 4 electrons per O_2 molecule), and F is the Faraday constant.

Supplementary Tables

Table S1 Comparison of bifunctional catalytic activity of catalysts at a current density of 100 mA cm^{-2} and specific capacitance and ECSA for all patterns in 1 M KOH.

Catalysts	Overpotential $_{HER}$(mV)	Overpotential $_{OER}$(mV)	C_{dl}(mF cm^{-2})	ECSA($cm^{-2}{}_{ECSA}$)
BS-1 hybrid	158	296	44.5	1112.5
BS-2 hybrid	183	323	33.8	845
BS-3 hybrid	187	330	31	775
BW-1 hybrid	190	311	37.6	940
BW-2 hybrid	167	305	42.4	1060
BW-3 hybrid	192	299	38.9	972.5
R-P phosphide	322	378	8.8	220
NW phosphide	342	352	21.6	540
NF metal	374	--	1	25

Note: --indicates that the catalytic activity of catalyst NF did not reach 100 mA cm^{-2}.

Table S2 Comparison of the overall water splitting performance of BS-1 with reported high-performance bifunctional catalysts in 1 M KOH electrolyte at 25 °C.

**Bifunctional electrocatalysts**	**Cell voltage (V) at 20 mA cm^{-2}**	**Reference**
BS-1	1.63	This work
FeP/Ni_2P	1.50	Nat. Commun., 2018, 9, 2551
Ir-NSG	1.53	Nat. Commun., 2020, 11, 4246
SLC	1.53	Appl Catal B-Environ, 2021, 299, 120658
Fe-CoP/NF	~1.54	Adv. Sci., 2018, 1800949
MoS_2-AB/NF	1.58	Nano Energy, 2022, 92, 106707
CoMnO@CN	1.60	J. Am. Chem. Soc., 2015, 137, 45, 14305−14312
Mo_2NiB_2	1.62	Small, 2021, 2104303
Ni-ZIF/Ni-B@NF-4	1.63	Adv. Energy Mater., 2019, 1902714
ZIF@LDH@Ni foam-600	1.63	ACS Appl. Mater. Inter, 2017, 9, 42, 36762−36771
$Co_{0.2}$-VOOH	1.64	Nanoscale, 2019,11, 18238-18245
NiFeOH/CoS_x/NF	1.66	J. Mater. Chem. A, 2020, 8, 13795−13805
NiFe LDH@NiCoP/NF	1.67	Adv. Funct. Mater., 2018, 28, 1706847
O-NiMoP/NF	~1.71	Adv. Funct. Mater., 2021, 31, 2104951
CoP NFs	1.72	ACS Catal., 2020, 10, 412−419
Co_2P	1.74	J Power Sources, 2018, 402, 345-352

Note: ~ indicates valuation in the literature.

Table S3 Comparison of overall water splitting activities at industrial conditions and stability test.

Type	Electrodes	Electrolyte	Temperature (°C)	Performance	Stability time	Reference
Zero-gap	BS-1$^{(+)}$‖BS-1$^{(–)}$	6 M KOH	85	2.25 V<br>3.5 A/cm^2	2 A/cm^2<br>330 h	This work
AEM	$IrO_2$$^{(+)}$‖Pt/C$^{(–)}$	PFTP-8/PFBP-14, 1 M KOH	80	2 V<br>7.68 A/cm^2	--	Energy Environ. Sci., 2021, 14, 6338-6348
AEM	NiFe$^{(+)}$‖PtRu$^{(–)}$	HTMA-DAPP, 1 M NaOH	80	1.8 V<br>~4.85 A/cm^2	0.2 A/cm^2<br>~10 h	Nat Energy,<br>2020, 5, 378-385
AEM	Fe-NiMo-NH_3/$H_2$$^{(+)}$‖ NiMo-$NH_3$/$H_2$$^{(–)}$	X37-50 grade T, 1 M KOH	80	1.57 V<br>2.8 A/cm^2	--	Adv. Energy Mater., 2020, 10, 2002285
AEM	RANEY®-type-NiMo$^{(+)}$‖RANEY®-type-Ni$^{(–)}$	40 µm *m*-PBI, 24wt% KOH	80	2 V<br>2.8 A/cm^2	--	Energy Environ. Sci., 2019, 12, 3313
AEM	$IrO_2$$^{(+)}$‖$RuSe_2$$^{(–)}$	1 M KOH	80	1.8 V<br>1.77 A/cm^2	--	Small,<br>2021, 17, 2007333
AEM	NiFe-LDH/KB$^{(+)}$‖Pt/C$^{(–)}$	X37-50 grade T, 1 M KOH	80	1.59 V<br>1 A/cm^2	--	ACS Catal.,<br>2020, 10, 1886-1893
AEM	NiCoP@NiFeP$^{(+)}$‖NiCoP@NiFeP$^{(–)}$	1 M KOH	25	1.93 V<br>1 A/cm^2	1 A/cm^2<br>100 h	J. Mater. Chem. A, 2022, Doi:10.1039/D2TA01233B
AEM	20Ru/OMGC$^{(+)}$‖Ru-based$^{(–)}$	polymer membrane (X37-50gradeT, Dioxidematerial)	25	2 V<br>0.75 A/cm^2	--	Angew. Chem. Int., 2021, 60, 1441–1449
AEM	NiFe-BTC-GNPs$^{(+)}$‖ $MoNi_4$/$MoO_2$$^{(–)}$	ultrapure water	70	2 V<br>~0.68 A/cm^2	~0.54 A/cm^2 48 h	Energy Environ. Sci., 2020,13, 3447-3458
AEM	$IrO_2$$^{(+)}$‖Pt black$^{(–)}$	A-201, Tokuyama, deionized water	50	1.80 V<br>0.399 A/cm^2	0.2 A/cm^2<br>>535 h	J. Am. Chem. Soc., 2012, 134, 9054-9057
PEM	IrO_2@$TiO_2$$^{(+)}$‖Pt/C$^{(–)}$	Nafion 212	80	1.96 V<br>5 A/cm^2	--	Appl Catal B-Environ,<br>2020, 269, 118762
PEM	Nb/Ti/ss-PTL$^{(+)}$‖ss-	CCM with	80	1.91 V	--	Energy Environ. Sci.,

	mesh/carbon paper	Nafion 212		4 A/cm^2		2022, 15, 109-122
PEM	PSL$^{(+)}$||PSL/mesh-PTL$^{(-)}$	Nafion 115	90	2 V<br>2.5 A/cm^2	--	Adv. Energy Mater., 2021, 11, 2100630
PEM	60wt% Pt/C$^{(+)}$||1wt% $IrO_2$$^{(-)}$	sulfonated poly (phenylene sulfone)	80	2 V<br>~1.6 A/cm^2	1 A/cm^2<br>80 h	Adv. Energy Mater., 2020, 10, 1903995
		Nafion		2 V<br>~1.675 A/cm^2	1 A/cm^2<br>80 h	
PEM	IrO_x-Ir$^{(+)}$||Pt/C$^{(-)}$	Nafion 212	80	1.69 V<br>1.4 A/cm^2	2 A/cm^2<br>100 h	Angew. Chem., 2016, 128, 752-756
PEM	Pt/Ti$^{(+)}$||Au/carbon paper$^{(-)}$	commercial MEA with CSN115	67	1.75 V<br>1 A/cm^2	1.2 A/cm^2<br>200 h	J Power Sources, 2016, 307, 815-825
PEM	$Ir_{0.7}Ru_{0.3}O_x$(EC)$^{(+)}$||40 wt% Pt/C$^{(-)}$	Nafion 212 CS	80	1.69 V<br>1 A/cm^2	1 A/cm^2<br>400 h	Nano Energy, 2017, 34, 385-391
PEM	$IrO_2$$^{(+)}$\|\|3-DOM TIP-ITO$^{(-)}$	Aquivion™ E79-05S	130	1.68 V<br>0.9 A/cm^2	0.35 A/cm^2<br>1150 h	Energy Environ. Sci., 2014, 7, 820-830
PEM	$IrO_2$$^{(+)}$\|\|$RuTe_2$-M$^{(-)}$	water	80	1.8 V<br>0.67 A/cm^2	--	Small, 2021, 17, 2007333
PEM	YZRO/AB$^{(+)}$||Pt/C $^{(-)}$	Nafion 212	25	1.8 V<br>0.64 A/cm^2	--	Appl Catal B-Environ, 2019, 244, 494-501
Zero-gap	Ni foam$^{(+)}$||Ni perforated plate$^{(-)}$	30wt% KOH	70	~2.1 V<br>0.5 A/cm^2	--	J Environ Chem Eng, 2022, 10 107648
Zero-gap	NiFe$^{(+)}$||Ni foam$^{(-)}$	10wt% KOH	25	2 V<br>0.3 A/cm^2	~0.15 A/cm^2<br>10 h	Electrocatalysis, 2022. Doi: 10.1007/s12678-022-00734-6

Note: -- indicates not given and ~ indicates valuation in the literature; $^{(+)}$ and $^{(-)}$ are used as anode and cathode in the electrolyzer, respectively.

Supplementary Figures

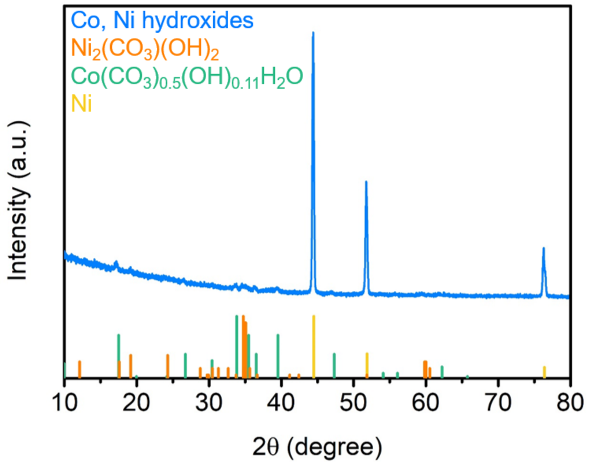

Figure S1. XRD patterns of as-prepared Co-Ni hydroxide on Ni sheet.

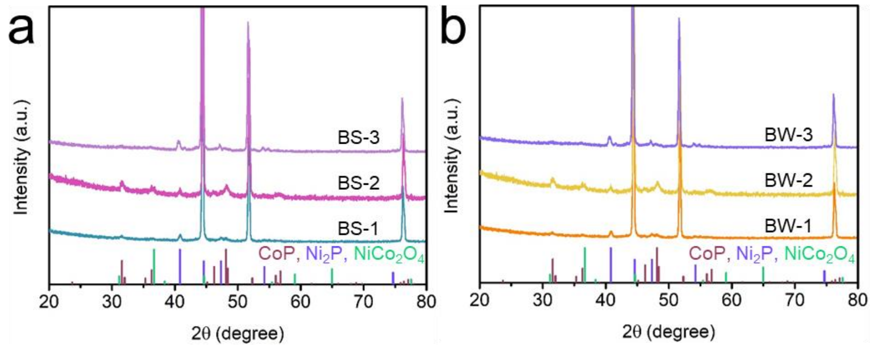

Figure S2. XRD patterns of (a) sheet bundles for BS-1, BS-2 and BS-3; (b) wire bundles for BW-1, BW-2 and BW-3.

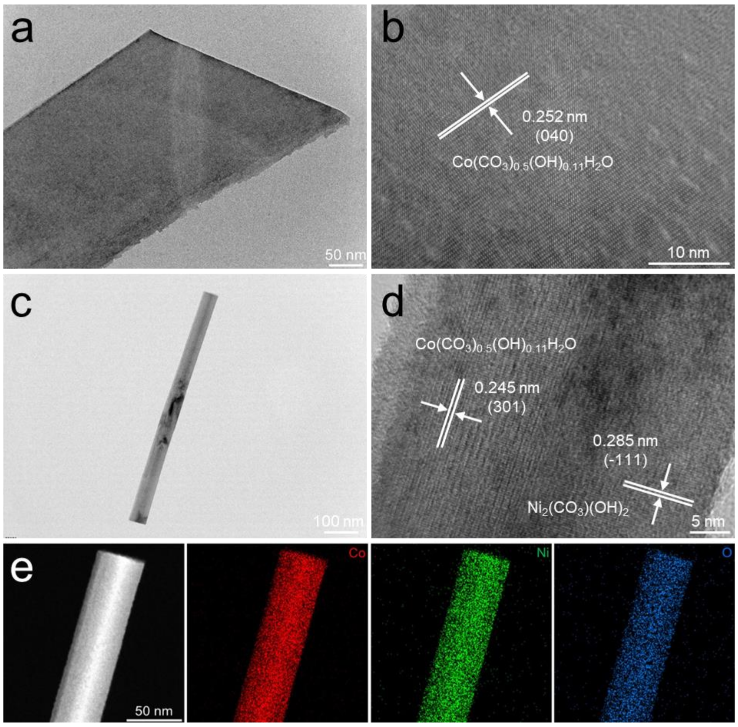

Figure S3. (a) TEM image and (b) HRTEM image of the sheet in the precursor Co-Ni hydroxides; (c) TEM, (d) HRTEM and (e) HAADF images of the rod in the precursor Co-Ni hydroxides.

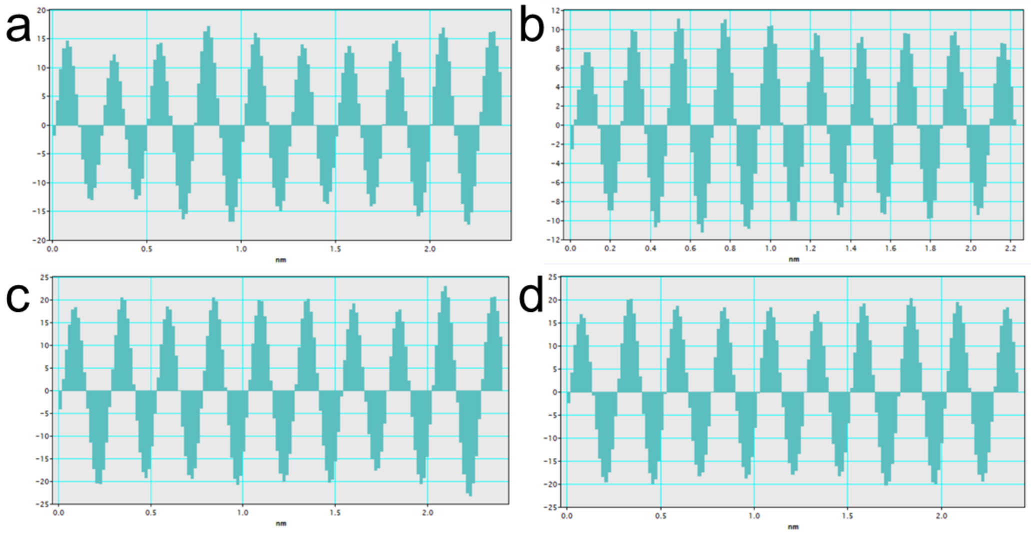

Figure S4. The intensity distribution of the Gatan digital micrograph corresponds to the lattice spacing of the enlarged part in Figure 1d, d-spacings intensity of (a) CoP, (b) Ni_2P, (c) (d) $NiCo_2O_4$.

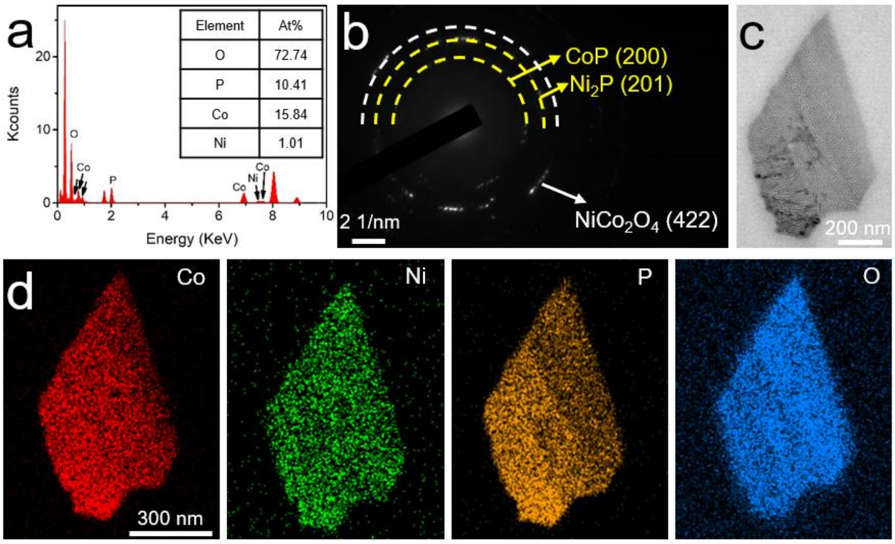

Figure S5. (a) The EDX spectrum of BS-1, the atomic ratio of each element is shown in the embedded table; (b) SAED patterns, (c) TEM image and (d) EDX element mappings of Co, Ni, P, O elements for BS-1.

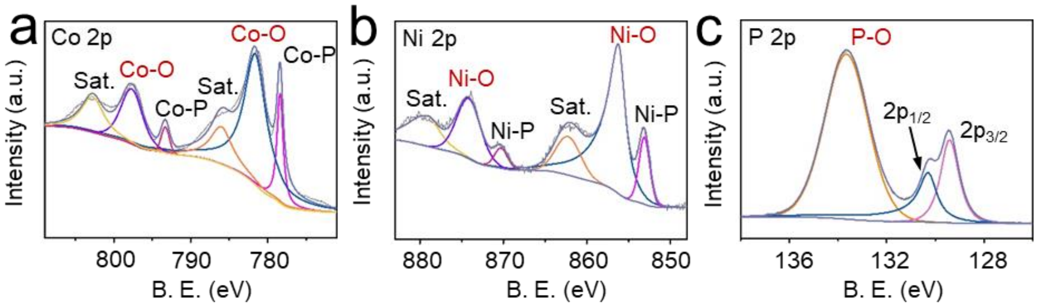

Figure S6. High-resolution XPS spectra of (a) Co, (b) Ni and (c) P for BS-1.

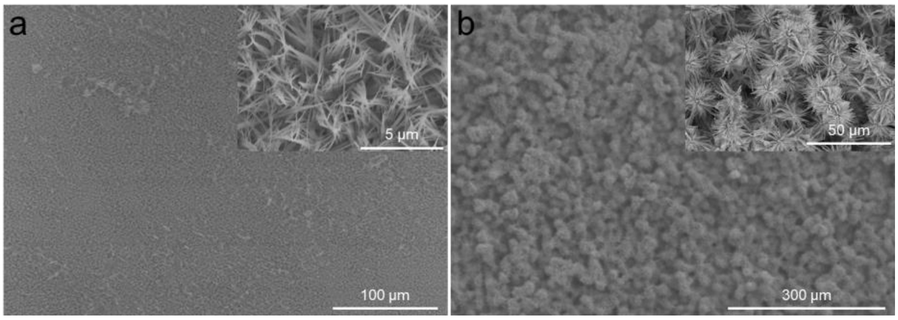

Figure S7. SEM images of precursor Co-Ni hydroxides with (a) nanowires and (b) clusters.

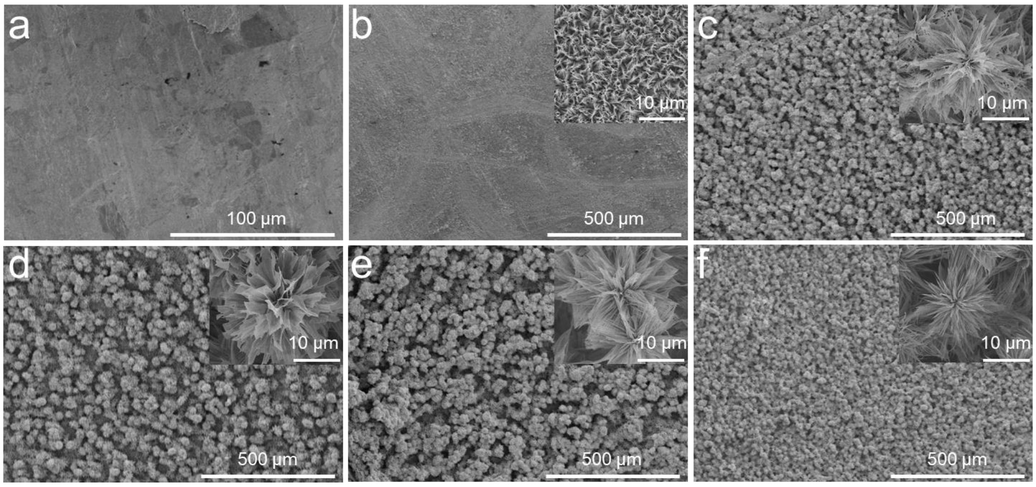

Figure S8. SEM images of (a) blank Ni sheet; (b) phosphide nanowires (NW); sheet bundles (c) BS-2 and (d) BS-3; wire bundles (e) BW-2 and (f) BW-3.

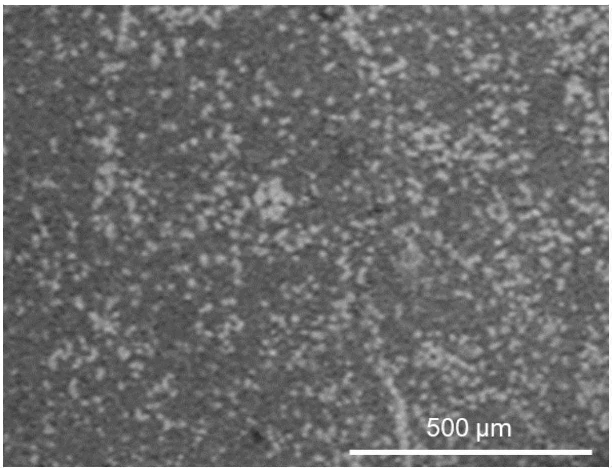

Figure S9. SEM image of regular phosphide surface without pattern (R-P).

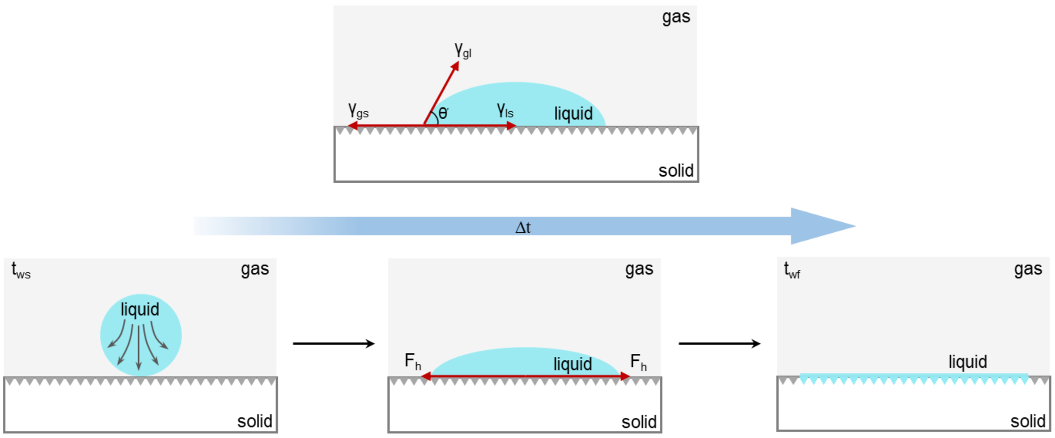

Figure S10. The schematic diagram of the surface tension of the gas-liquid-solid three-phase interface (top); the schematic diagrams showing t_{ws}, t_{wf} and Δt during the spreading of a water droplet on the superhydrophilic surface.

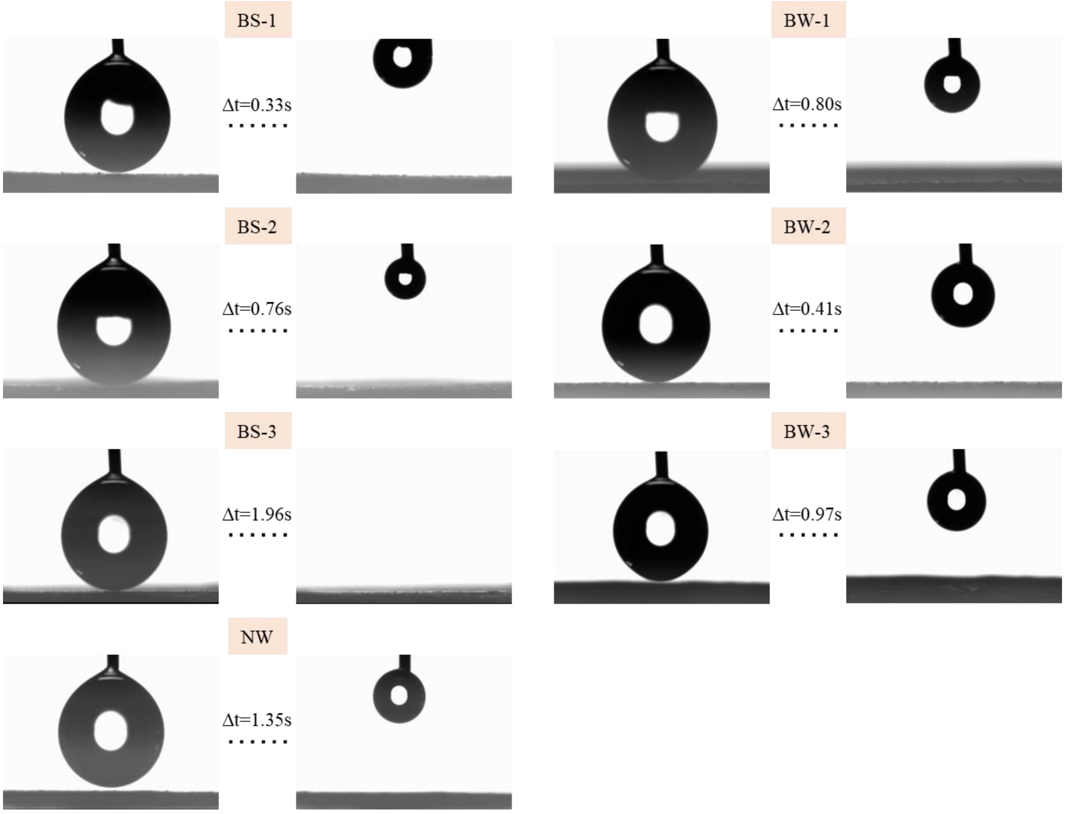

Figure S11. Snapshots from videos showing the spread time (Δt) of all the patterns surfaces.

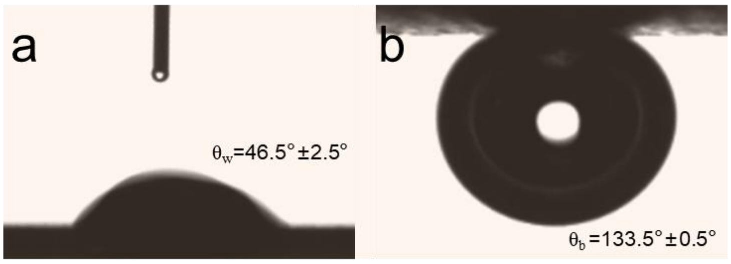

Figure S12. Optical images of (a) water and (b) air bubble on the surface of pressed NF.

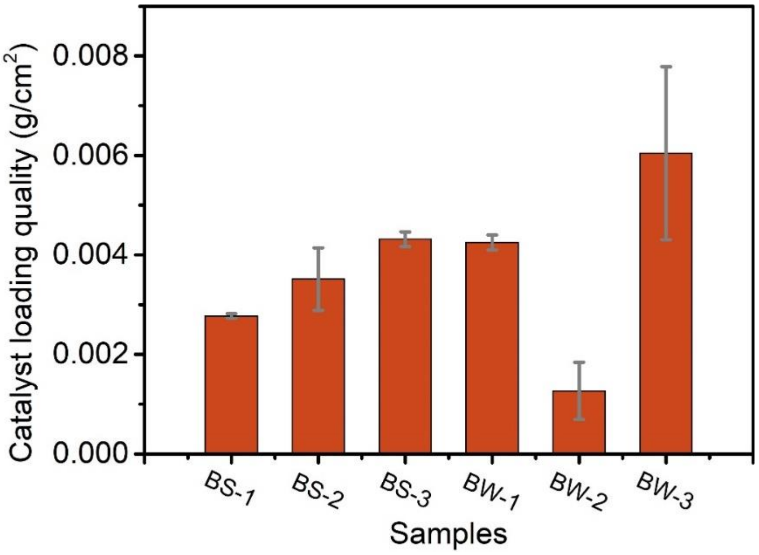

Figure S13. The catalyst loadings of various hybrid materials on NF.

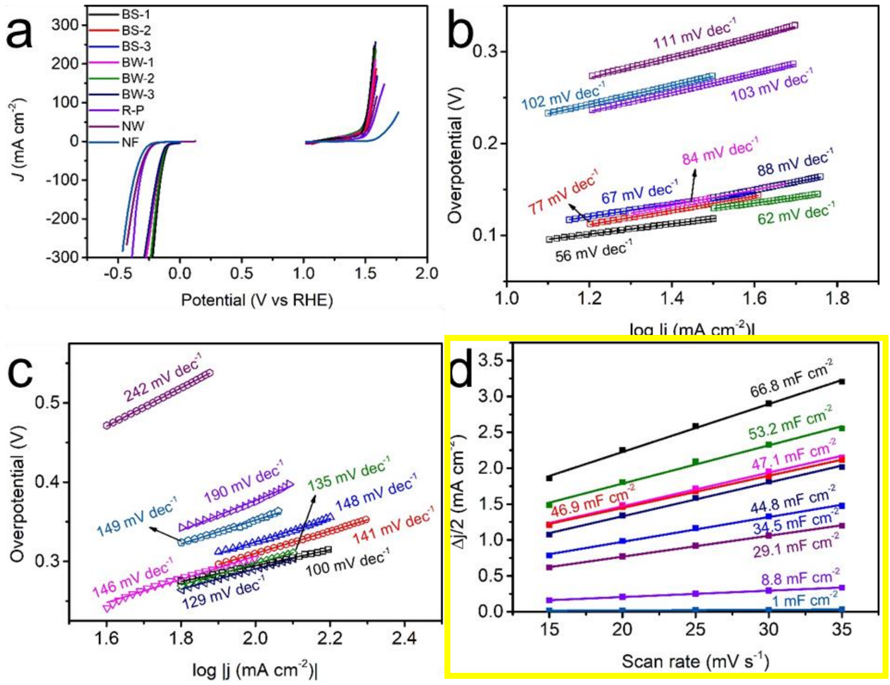

Figure S14. (a) Linear sweep voltammetry curves of HER and OER; the corresponding (b) Tafel slopes derived from the HER polarization curves and (c) Tafel slopes derived from the OER polarization curves; (d) electric double-layer capacitance (C_{dl}).

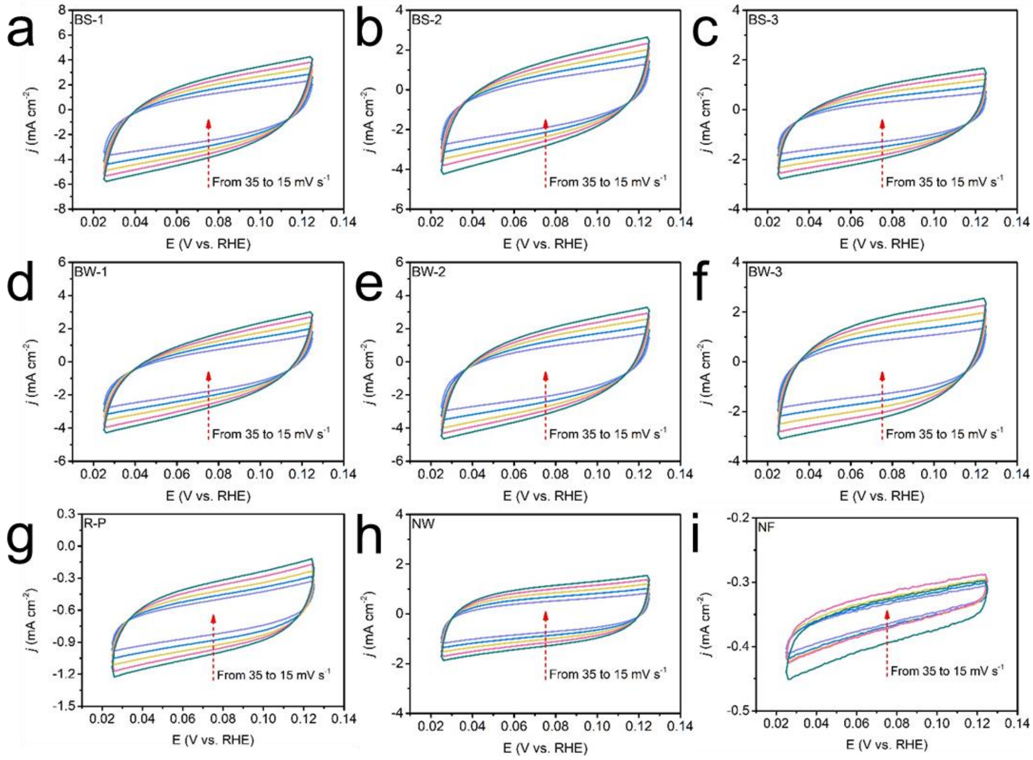

Figure S15. The CV curves of (a) BS-1, (b) BS-2, (c) BS-3, (d) BW-1, (e) BW-2, (f) BW-3, (g) R-P, (h) NW and (i) NF, which are used for ECSA calculation.

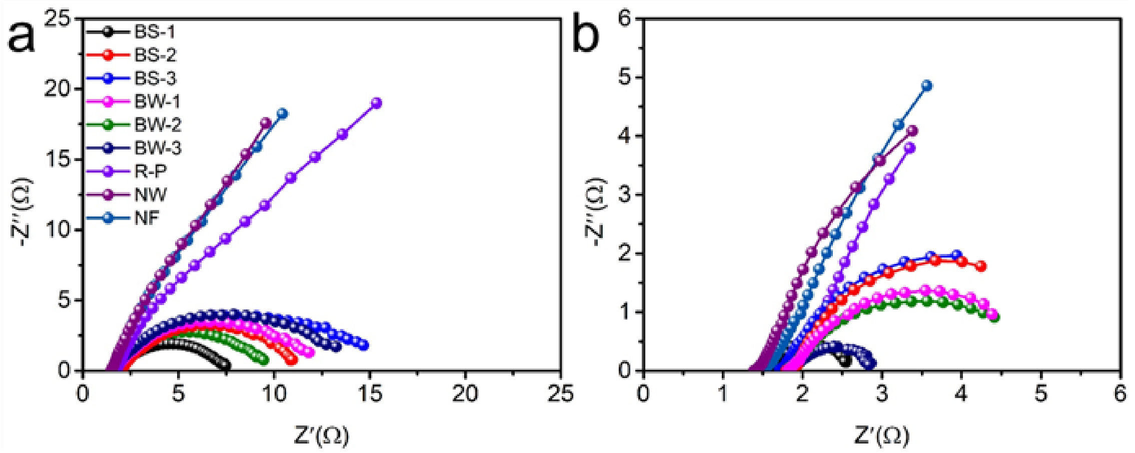

Figure S16. EIS plots of catalysts in (a) OER and (b) HER at the overpotential of 75 mV.

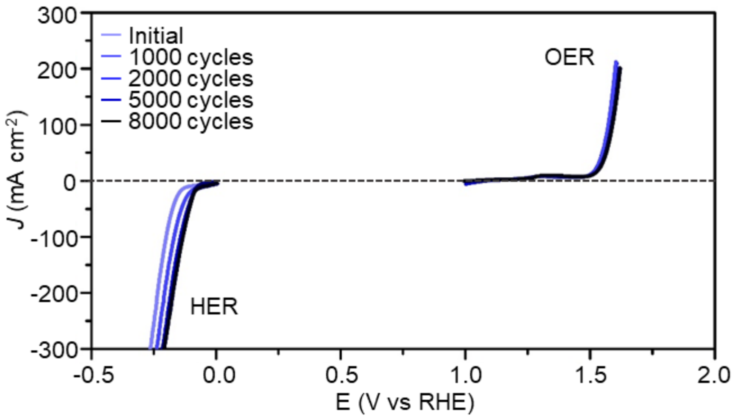

Figure S17. Cyclic voltammograms of BS-1 in OER and HER.

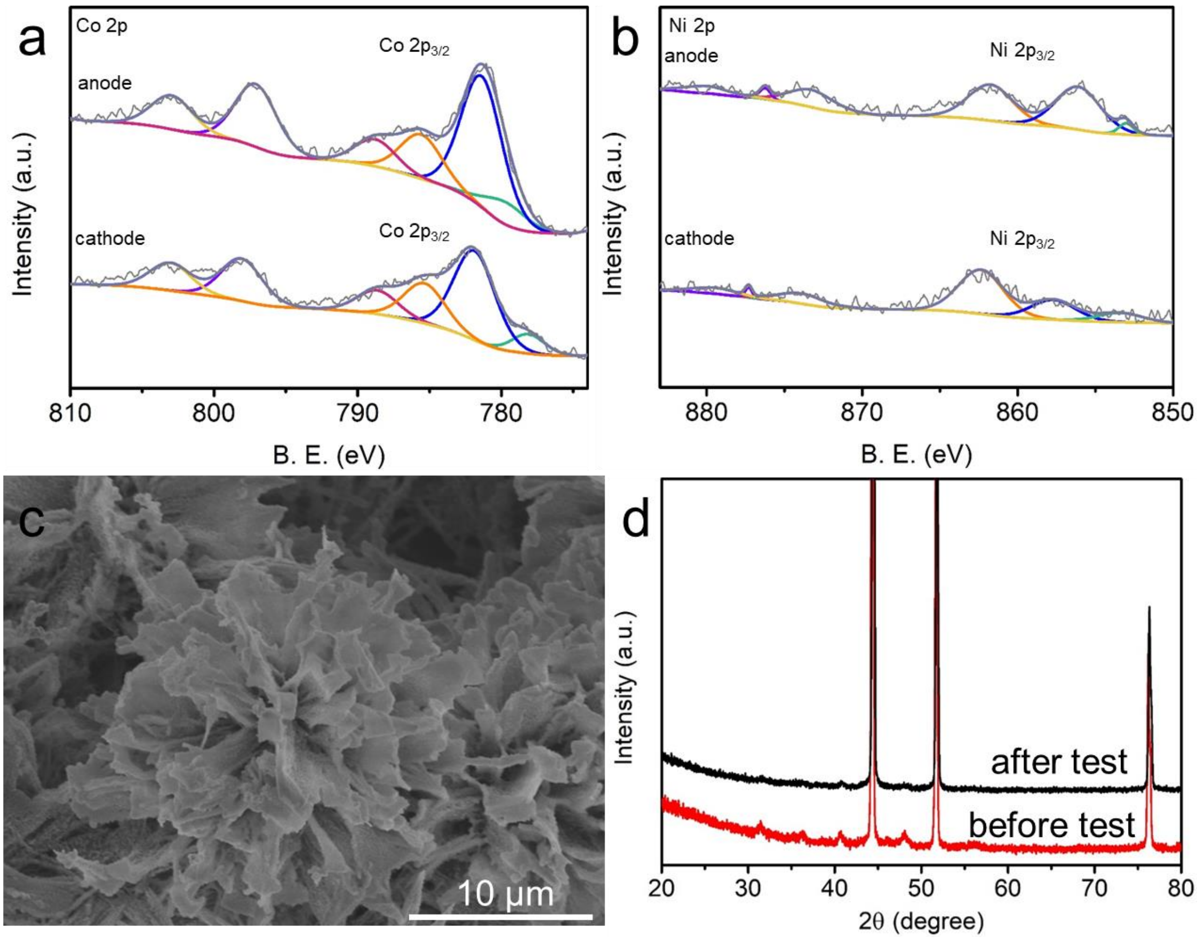

Figure S18. XPS spectra of (a) Co 2p and (b) Ni 2p core-level for the spent catalyst BS-1 after the stability test at 25 °C; (c) SEM image and (d) XRD patterns of the spent catalyst BS-1 after the stability test at 25 °C in Fig. 4e.

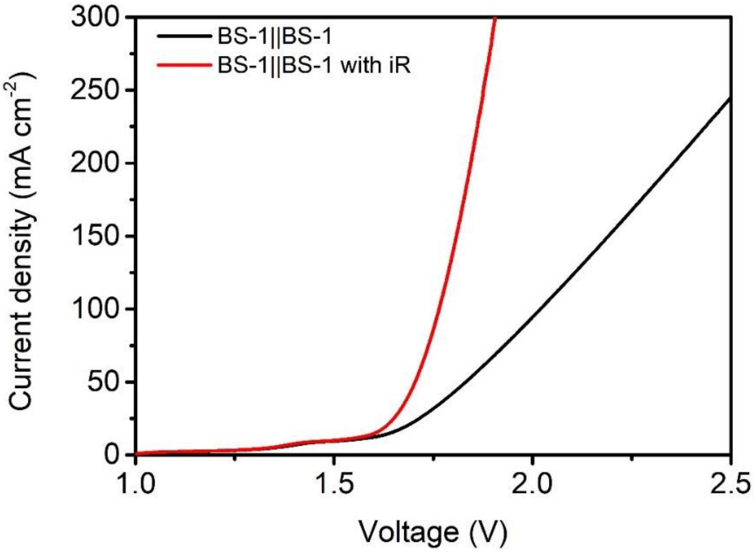

Figure S19. The polarization curves of a two-electrode water electrolyzer in 1 M KOH using BS-1 as both HER and OER catalysts with and without iR compensation.

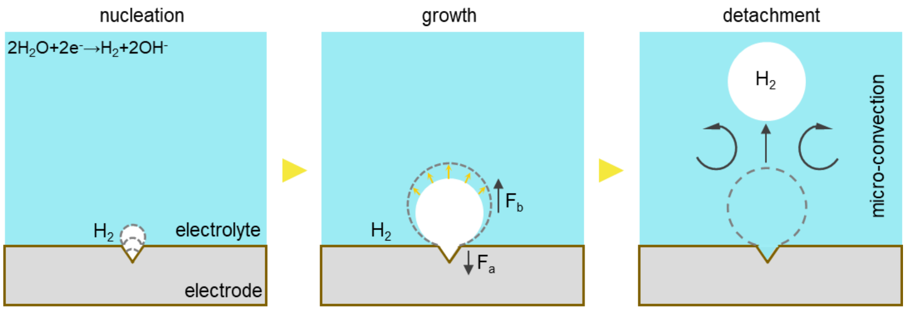

Figure S20. Various stages of bubble evolution during electrolysis, including nucleation, growth, and detachment of bubbles on the electrode surface.

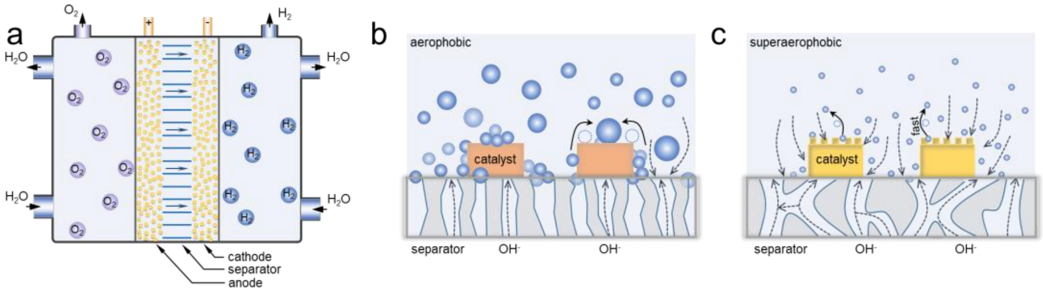

Figure S21. (a) Alkaline electrolyzer system with the zero-gap configuration. Schematic bubble evolution on (b) aerophobic and (c) superaerophobic electrode.

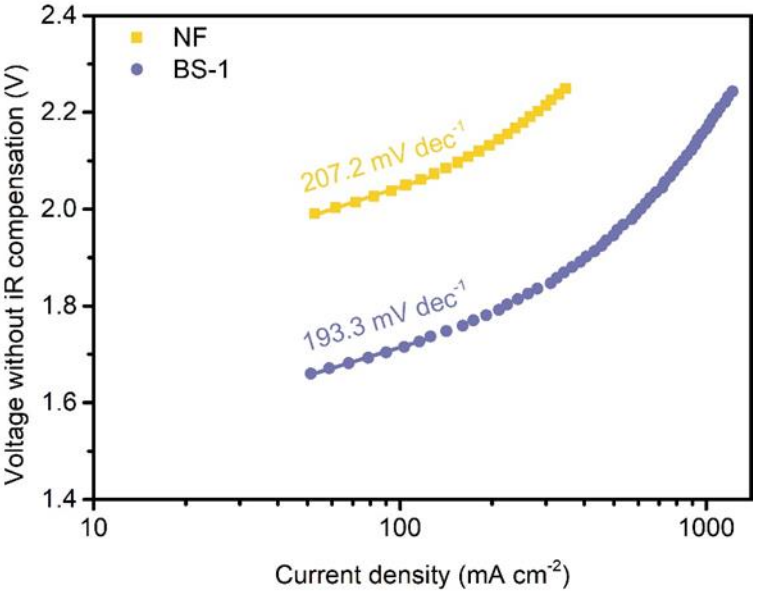

Figure S22. Tafel plot of the overall water electrolysis obtained from Fig. 4a for catalyst BS-1 and NF .

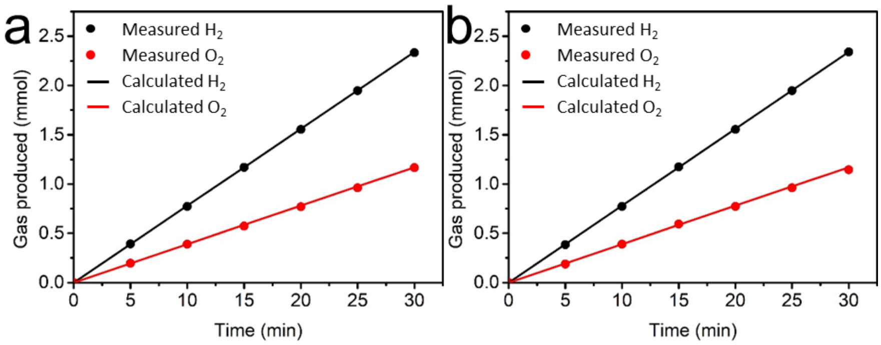

Figure S23. The amount of H_2 and O_2 collected as a function of time at (a) 25 ℃ and (b) 85 ℃.

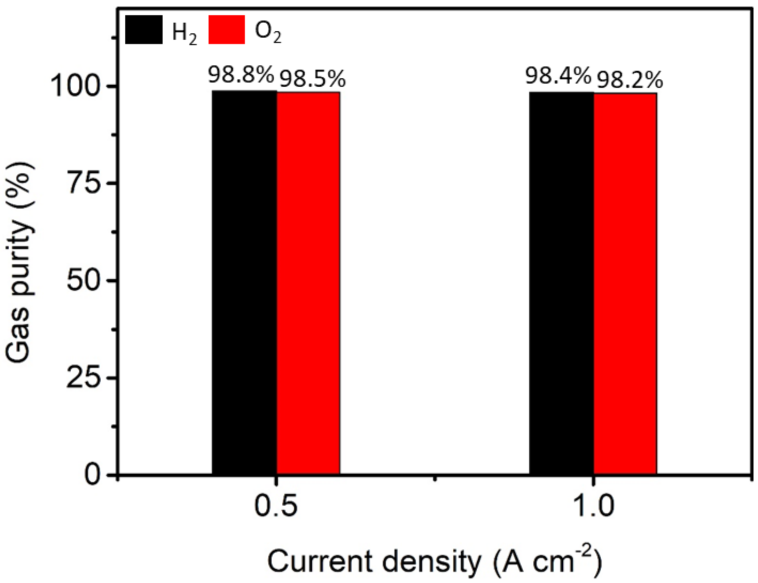

Figure S24. Gas purity of the effluent from the zero-gap electrolyser at 85 ℃.

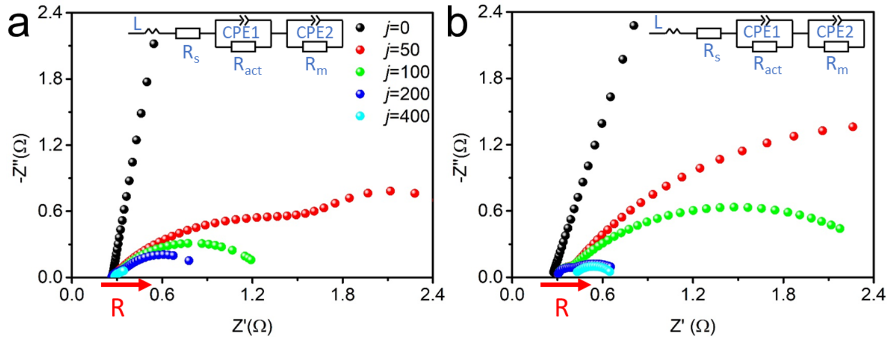

Figure S25. Comparison of the ohmic resistance for the zero-gap water elctrolyzer employed with (a) BS-1 and (b) NF electrode with current densities of 0, 50, 100, 200 and 400 mA cm^{-2}. Note the noisy signals in the low frequency regions have been removed.

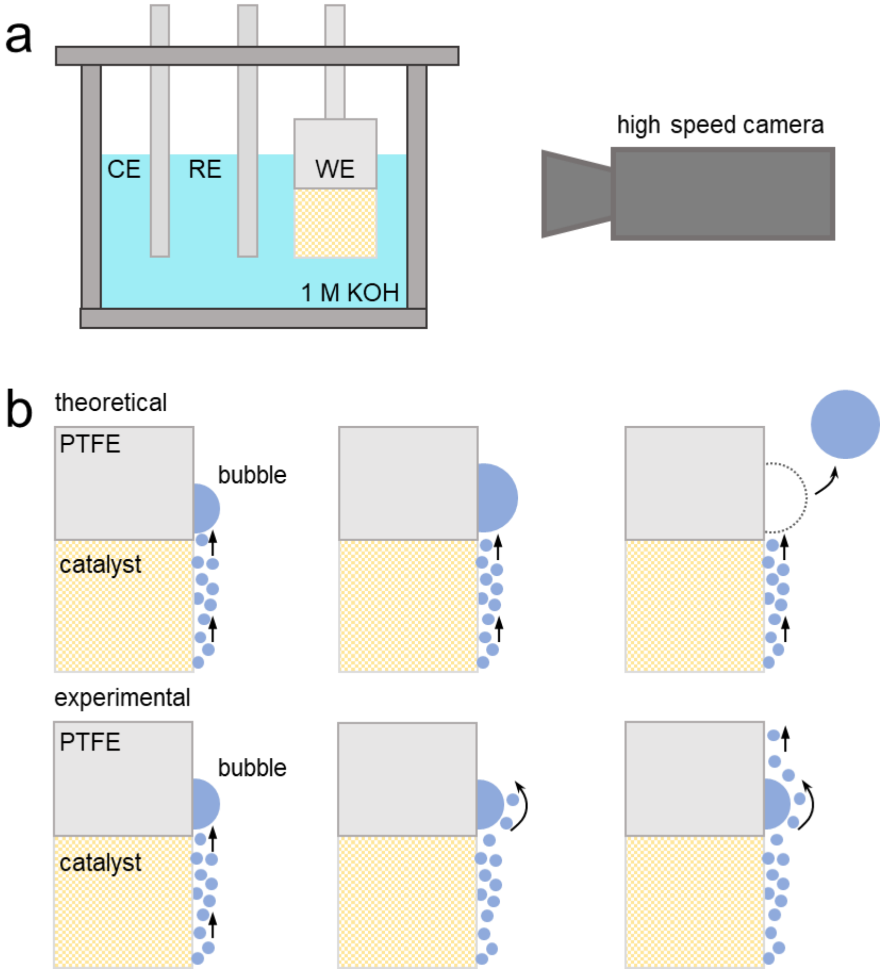

Figure S26. The setup to study the bubble behavior at the aerophobic/aerophilic interface using a high-speed camera. (a) the schematic structure of the setup; (b) the electrode with a PTFE and BS-1 domains. Theoretically, the bubble attached to the aerophilic PTFE domains would grow and the leave the surface. Yet experimentally we observe that such bubble is “permanent”, showing little dynamic behaviors. This is because the evolved small bubbles from BS-1 do not aggregate with the PTFE-attached bubble.

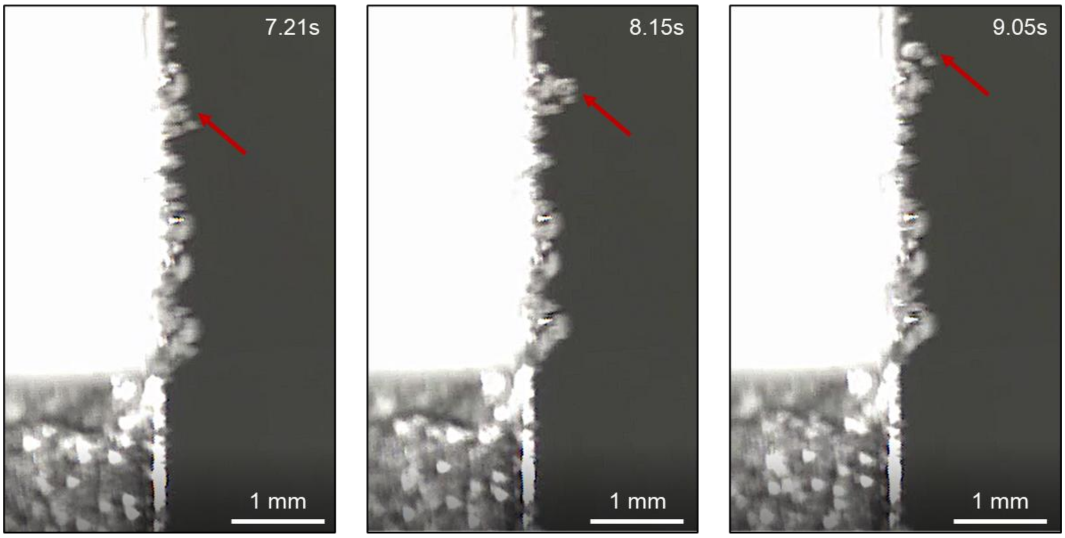

Figure S27. Bubble behaviors at the aerophobic/aerophilic interface from the high-speed camera videos.

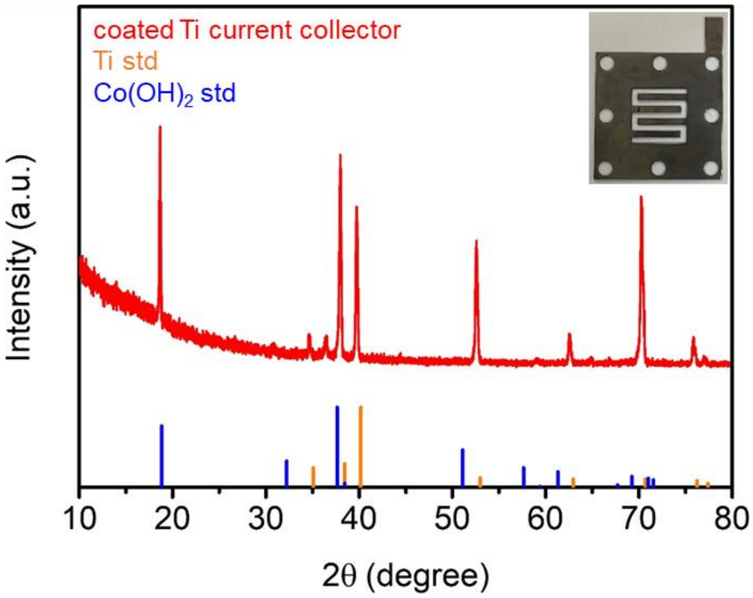

Figure S28. XRD curve and the photo (inset) of coated Ti current collector.

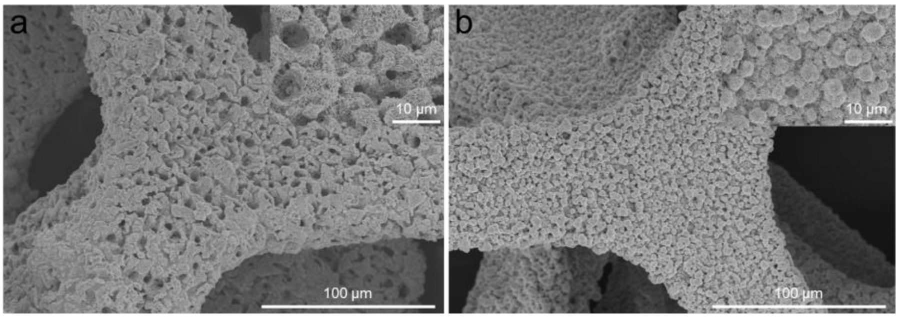

Figure S29. Morphologies of BS-1 electrodes in (a) HER and (b) OER after the 330 h longevity test in the zero-gap electrolyzer at 85 ℃.

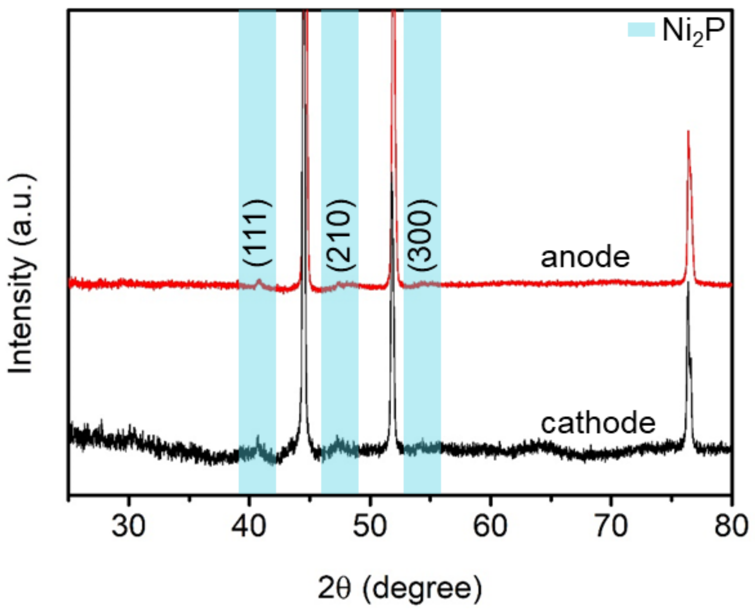

Figure S30. XRD patterns of BS-1 after the 330 h longevity test in the zero-gap electrolyzer at 85 ℃.

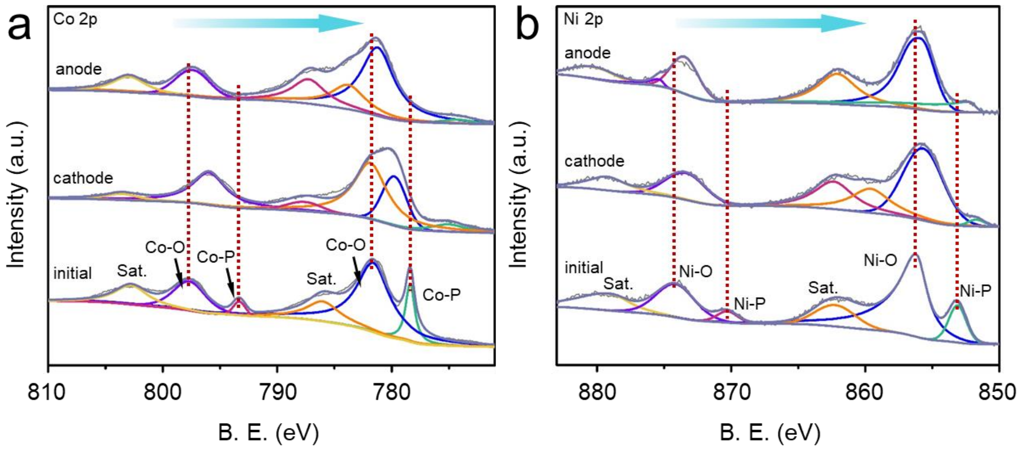

Figure S31. XPS spectra for BS-1 before and after 330 h test. High-resolution spectra for (a) Co 2p and (b) Ni 2p.
